# Concentration dependence of resistance components in solutions containing dissolved Fe^2+^/Fe^3+^

**DOI:** 10.1039/d3ra07829a

**Published:** 2024-02-20

**Authors:** Dai Inoue, Yutaka Moritomo

**Affiliations:** a Graduate School of Pure & Applied Science, University of Tsukuba Tennodai 1-1-1, Tsukuba Ibaraki 305-8571 Japan moritomo.yutaka.gf@u.tsukba.ac.jp; b Faculty of Pure & Applied Science, University of Tsukuba Tennodai 1-1-1, Tsukuba Ibaraki 305-8571 Japan; c Tsukuba Research Center for Energy Materials Science (TREMS), University of Tsukuba Tsukuba Ibaraki 305-8571 Japan

## Abstract

Electrolyte solutions containing Fe^2+^/Fe^3+^ are suitable for liquid thermoelectric conversion devices (LTEs), because they are inexpensive materials and exhibit a high electrochemical Seebeck coefficient *α*. Here, we investigated the concentration (*c*) dependence of resistance components, *i.e.*, solvent (*R*_s_), charge-transfer (*R*_ct_), and diffusion (*R*_dif_) resistances, of dissolved-Fe^2+^/Fe^3+^-containing aqueous, methanol (MeOH), acetone, and propylene carbonate (PC) solutions. We found that the *c* dependence of *R*_s_ and *R*_dif_ are well reproduced by empirical formulas, 
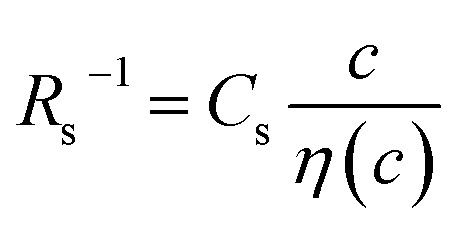
 and 
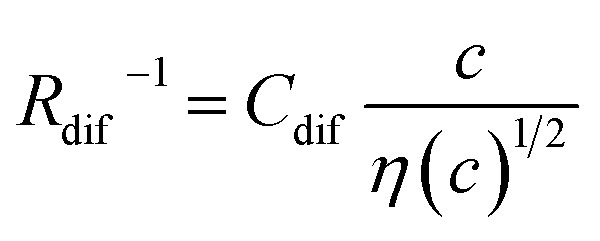
, where *η*(*c*) is viscosity at *c*. We further found that the magnitudes of *C*_s_ and *C*_dif_ are nearly independent of solvent, suggesting that *η* is one of the significant solution parameters that determine *R*_s_ and *R*_dif_.

## Abstract 2

The concentration (*c*) dependence of solution (*R*_s_) and diffusion (*R*_dif_) resistances of solutions containing dissolved Fe^2+^/Fe^3+^ is well reproduced by their viscosity *η*(*c*) at *c*.

## Graphical abstract

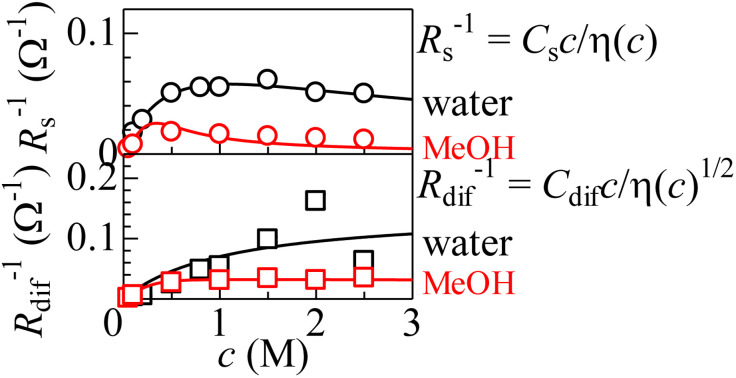

## 1 Introduction

Energy-harvesting devices are attracting the current attention of researchers from the viewpoint of a basic power source for the internet of things (IoT) society as well as sustainable development goals (SDGs). Thermoelectric conversion devices (TEs) are promising because they cover the field from bioelectric and wearable electronics to industrial power generation. In particular, flexible TEs using cellulose gel^1^ have excellent compatibility with various thermoelectric conversion materials. This is because cellulose can be used in the design of flexible and ion/electron conductive materials with robust mechanical properties. Flexible TEs are expected to be used not only for thermoelectric conversion but also for sensors and refrigeration units.

Among TEs, liquid thermoelectric conversion devices (LTEs) are promising because they are made of inexpensive materials. There is already a long history of LTE research.^2^ Nevertheless, vigorous research has become more active in recent years and many research results have been reported.^3–19^ The performance of LTEs is governed by the electrochemical Seebeck coefficient *α*, effective electric conductivity *σ*, and effective thermal conductivity *κ* of the electrolyte.^20^ Unlike solid thermoelectric devices, *σ* is related to the charge transfer and diffusion processes of redox ions as well as the conventional ion migration. The magnitude of *σ* depends on the microscopic structure and material of the electrodes.^16,17^ In addition, effective *σ* and *κ* are influenced by convection of the electrolyte induced by Δ*T*. The dimensionless figure of merit (
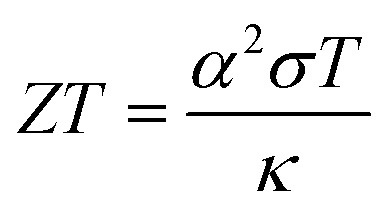
, where *T* is temperature) is a measure of the LTE performance. With the increase of *ZT*, the thermal efficiency *η* increases toward the Carnot efficiency 
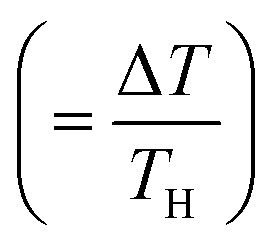
, which is the maximum efficiency of a heat engine.^21^ To enhance *ZT*, it is effective to increase (decrease) *α* and *σ* (*κ*).

In recent years, research on LTEs using an organic electrolyte^18,19^ has begun to attract attention, as has research on conventional LTEs using an aqueous electrolyte.^4–17^ This is because the organic electrolytes exhibit both large *α* and small *κ*. In several organic solutions containing Fe^2+^/Fe^3+^, *α* is higher than the value (= 1.4 mV K^−1^) of an aqueous solution. For example, *α* is 3.6 mV K^−1^ in acetone solution and 1.8 mV K^−1^ in propylene carbonate (PC) solution.^22^ In addition, *κ* of a typical organic solvent is ≈ 0.2 W K^−1^ m^−1^ and is approximately 33% of the value (= 0.6 W K^−1^ m^−1^) of water. Recently, Wake *et al.*^18,19^ showed that LTEs composed of dissolved-Fe^2+^/Fe^3+^-containing methanol (MeOH) and acetone solutions exhibit a large power factor (PF = *α*^2^*σ*) comparable to that of the corresponding aqueous LTE. They also reported *α* and *σ* against solute concentration *c*. The disadvantage of organic electrolytes is a small *σ* value compared with that of an aqueous electrolyte. Except for aqueous electrolyte,^7^ there exists no detailed investigation on the resistance components. Therefore, the origin of the small *σ* in organic electrolytes is still unclear. Here, we will investigate the resistance components of several solutions containing dissolved Fe^2+^/Fe^3+^ against *c* to deeply understand *σ* and to obtain guidelines for increasing *σ* in organic electrolytes.

In general, the resistance *R* (
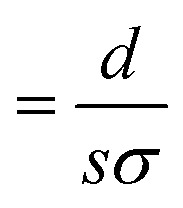
, where *d* and *s* are the electrode distance and area, respectively) of an electrolyte solution consists of the solution resistance *R*_s_ due to ion migration, charge transfer resistance *R*_ct_ due to electron transfer, and diffusion resistance *R*_dif_ due to reactant/product diffusion.^23^ Among them, *R*_s_ is derived from the balance between the electric force (=|*z*|*eE*_ef_; |*z*|, *e*, and *E*_ef_ are the charge number, elementary charge, and electric field, respectively) and frictional force. According to Stokes’ law, the latter force is expressed as 6π*ηrv*, where *η*, *r*, and *v* are the viscosity, effective radius, and velocity of the ion, respectively. Then, the mobility *u*
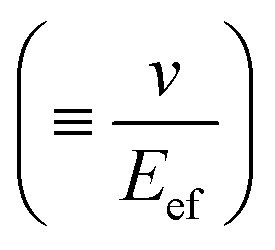
 of an ion is expressed as 
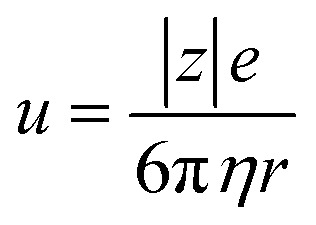
 and is inversely proportional to *η*. On the other hand, *R*_ct_ and *R*_dif_ are governed by the reaction kinetics in the vicinity of the electrode surface and are independent of *d*. The reaction rate *k* is expressed as 
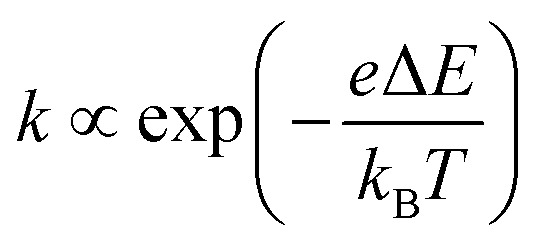
, where Δ*E* (= *E* − *E*_eq_; *E* and *E*_eq_ are the electrode and equilibrium potentials, respectively) and *k*_B_ are the overpotential and Boltzmann constant, respectively. In the region of 
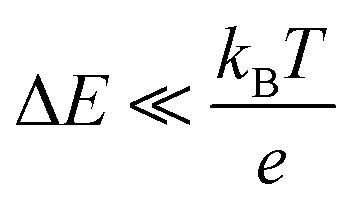
, the charge-transfer current *J*_ct_ is expressed as 
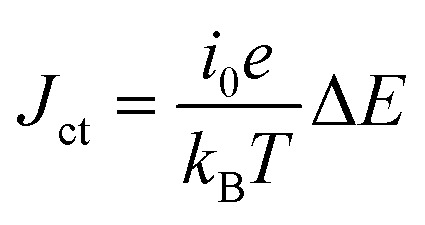
,^23^ where *i*_0_ is the exchange current. Thus, *R*_ct_ is proportional to 
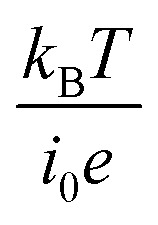
 and is independent of *d*. The physical meaning of *R*_dif_ is as follows. As the reaction progresses, the concentration of reactants/products at the electrode surface changes in a way that prevents further reaction. For the reaction to continue, the reactants/products must diffuse into/from the bulk region. Note that the diffusion current of reactants/products is driven by the concentration gradient created by the reaction at the electrode surface and is independent of *d*.

In this work, we investigated the *c*-dependence of *R*_s_, *R*_ct_, and *R*_dif_ of dissolved-Fe^2+^/Fe^3+^-containing aqueous, MeOH, acetone, and PC solutions. We found that the *c*-dependence of *R*_s_ and *R*_dif_ is well reproduced by empirical formulas, 
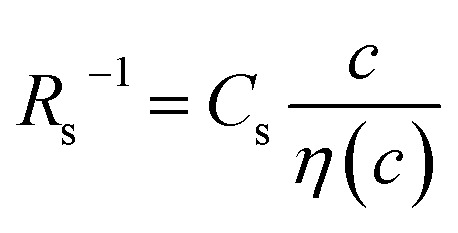
 and 
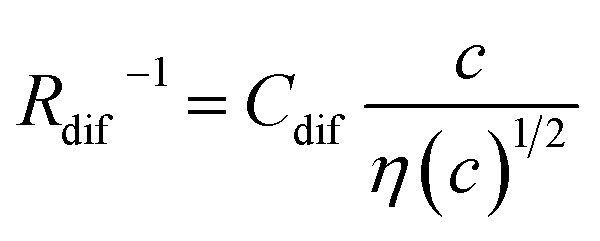
. We further found that their coefficients, *C*_s_ and *C*_dif_, are nearly independent of solvent, suggesting that *η* is one of the significant solution parameters that determine *R*_s_ and *R*_dif_.

## 2 Experimental methods

### 2.1 Solution preparation

In this study, water, MeOH, acetone, and PC were selected as the solvents because they exhibit a high solubility of Fe(ClO_4_)_2_/Fe(ClO_4_)_3_. We prepared aqueous, MeOH, acetone, and PC solutions containing *c* M Fe(ClO_4_)_2_·6.0H_2_O and *c* M Fe(ClO_4_)_3_·7.1H_2_O. Distilled water, MeOH, acetone, PC, and solutes were purchased from FUJIFILM Wako Corp. and used as received. Table 1 shows the solubility *s* and critical concentration c* of Fe(ClO_4_)_2_/Fe(ClO_4_)_3_ in the four solvents. c* is defined as the concentration at which one Fe ion is dissolved per six solvent molecules. At *c* = c*, all solvent molecules are on average coordinated with Fe ions.

**Table 1:** Solubility *s* and critical concentration c* of Fe(ClO_4_)_2_·6.0H_2_O/Fe(ClO_4_)_3_·7.1H_2_O in several solvents at 298 K. c* is defined as the concentration at which one Fe ion is dissolved per six solvent molecules. MeOH and PC represent methanol and propylene carbonate, respectively

Solvent	*s* (M)	c* (M)
Water	2.5	4.62
MeOH	2.5	2.06
Acetone	1.2	1.12
PC	1.5	0.97

### 2.2 Total resistance

The total resistance *R*_tot_ of the electrolyte was measured in a two-pole cell at 298 K.^24^ The electrodes were produced from a 220 μm graphite sheet (PREMA-FOIL, TOYO TANSO). The electrode distance *d* and area *s* are 1.0 cm and 0.42 cm^2^, respectively. The voltage drop *V* was measured against the current *I* (*I* ≤ 0.4 mA) with a multimeter. *I* was changed in a stepwise manner at intervals of several minutes. *V* was stable and no change over time was observed. The slope of the *I*–*V* plot corresponds to *R*_tot_.

### 2.3 Electrochemical impedance spectroscopy

*R*_ct_ and *R*_dif_ were evaluated in the same cell with the same electrodes. *d* and *s* are 1.0 cm and 0.42 cm^2^, respectively. Electrochemical impedance spectroscopy (EIS) was performed at 298 K with use of a potentiostat (Vertex.one.EIS, Ivium Technologies). The frequency range was from 50 mHz to 100 kHz, and the amplitude was 10 mV. *V* was stable and no change over time was observed. It was confirmed that almost identical EIS data were obtained through multiple measurements.

The EIS data were analyzed with a Randles equivalent circuit,^23^ which consists of *R*_s_, *R*_ct_, double layer capacitance *C*_d_, and Warburg impedance *Z*_ω_. *Z*_ω_ is expressed as *Z*_ω_ = *A*_W_(*ω*^−1/2^ − i*ω*^−1/2^), where *A*_W_ and *ω* are the Warburg coefficient and angular velocity, respectively. It was difficult to evaluate the magnitude of *R*_dif_ from *A*_W_ even though *Z*_ω_ describes the diffusion process of the reactants/products. In the present study, we tentatively evaluate the *R*_dif_ values by subtraction of *R*_s_ and *R*_ct_ from *R*_tot_. We confirmed a positive correlation between *A*_W_ and *R*_dif_ (= *R*_tot_ − *R*_s_ − *R*_ct_), which strongly supports the correctness of our evaluation method of *R*_dif_ (*vide infra*).

## 3 Results

### 3.1 Total resistance

**Fig. 1:**
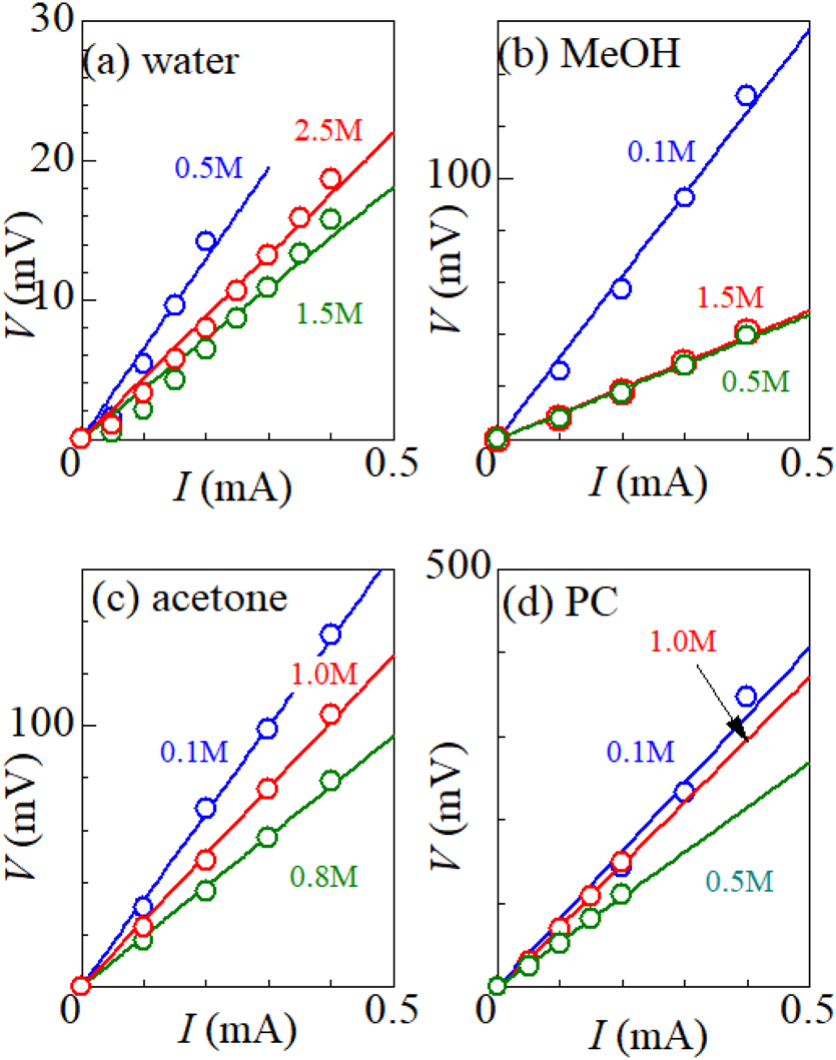
Voltage *V* against current *I* for several solutions containing dissolved Fe^2+^/Fe^3+^ at 298 K: (a) water, (b) MeOH, (c) acetone, and (d) PC. Data for three typical solute concentrations are shown for each solution: below the resistance minimum (blue), near the resistance minimum (green), and above the resistance minimum (red). The straight lines are the results of least-squares fits.

Fig. 1 shows examples of the *I*–*V* plot of several solutions containing dissolved Fe^2+^/Fe^3+^ at 298 K: (a) water, (b) MeOH, (c) acetone, and (d) PC. For all solutions, *V* increases in proportion to *I*. *R*_tot_ was evaluated from the slope of the plots, as indicated by the straight lines. The obtained *R*_tot_ values are listed in Table 2. In (a) the aqueous solution, *R*_tot_ decreases from 65.0 Ω at 0.5 M to 28.5 Ω at 1.5 M, and then increases to 37.9 Ω at 2.5 M. The decrease in *R*_tot_ in the low *c* region is due to the increase in the number (∝ *c*) of charge carriers, such as Fe^2+^ and Fe^3+^. This behavior is consistent with the literature.^6^ Similar local minima structures in the *c*–*R*_tot_ plot are also observed for the MeOH, acetone and PC solutions (Table 2).

**Table 2:** Total resistance *R*_tot_, solution resistance *R*_s_, charge-transfer resistance *R*_ct_, diffusion resistance *R*_dif_, and Warburg coefficient *A*_W_ in solvents containing *c* M Fe(ClO_4_)_2_ and *c* M Fe(ClO_4_)_3_. *R*_dif_ was evaluated by subtraction of *R*_s_ and *R*_ct_ from *R*_tot_

Solvent	*c* (M)	*R*_tot_ (Ω)	*R*_s_ (Ω)	*R*_ct_ (Ω)	*R*_dif_ (Ω)	*A*_W_ (Ω s^−1/2^)
Water	0.10	351.0	56.0	32.0	263.0	86.2
Water	0.20	236.0	35.4	16.1	184.5	42.8
Water	0.50	65.0	19.8	5.3	39.9	15.7
Water	0.80	42.6	18.1	3.8	20.7	10.8
Water	1.00	39.4	18.1	2.9	18.4	10.0
Water	1.50	28.5	16.3	2.1	10.1	9.1
Water	2.00	27.7	19.6	1.9	6.2	7.0
Water	2.50	37.9	20.0	1.9	16.0	10.4
MeOH	0.05	906.0	221.7	65.2	619.1	176.7
MeOH	0.10	314.0	126.0	22.6	165.4	63.0
MeOH	0.50	95.6	54.6	4.4	36.6	16.2
MeOH	1.00	95.2	60.9	1.8	32.5	9.1
MeOH	1.50	98.2	67.4	1.6	29.2	8.4
MeOH	2.00	110.0	75.5	2.4	32,1	6.8
MeOH	2.50	115.0	83.3	3.2	28.6	7.5
Acetone	0.05	692.0	439.2	35.6	227.2	134.6
Acetone	0.10	333.0	187.0	18.0	128.0	72.0
Acetone	0.20	225.0	130.4	8.6	86.0	29.0
Acetone	0.40	172.0	100.3	5.0	66.7	14.7
Acetone	0.80	192.0	126.8	2.8	62.4	9.0
Acetone	1.00	254.0	160.5	4.5	89.0	9.2
Acetone	1.20	251.0	180.0	4.5	66.5	8.8
PC	0.05	1265.0	726.8	75.6	425.6	255.8
PC	0.10	813.0	615.0	27.0	171.0	70.0
PC	0.20	528.0	387.6	11.5	128.9	31.2
PC	0.50	537.0	447.9	11.4	77.7	17.5
PC	1.00	741.0	663.3	12.9	64.8	15.0
PC	1.50	726.0	674.1	13.2	38.7	13.9

Let us estimate the maximum value of *ZT* in the aqueous electrolyte at 300 K with the use of *R*_tot_ shown in Table 2. The maximum value of *σ*
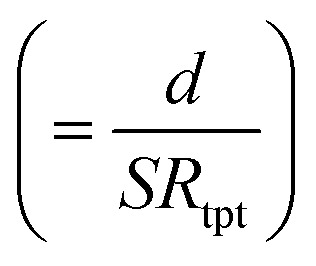
 is 86.0 mS cm^−1^ at *c* = 2.0 M. Kim *et al.*^6^ reported *c*-dependence of *α* and *κ* in an aqueous solution containing Fe(ClO_4_)_2_/Fe(ClO_4_)_3_. From the extrapolation of the reported data, we evaluated *α* = 1.76 mV K^−1^ and *κ* = 0.4 W K^−1^ m^−1^ at 2.0 M. Then, we obtained *ZT* = 0.020 at 2.0 M. The *ZT* value is smaller than the value (= 0.036 (ref. 6) at 0.8 M) reported by Kim *et al.*,^6^ reflecting the smaller *σ* obtained in the present experiment. We note that effective *σ* of a LTE is influenced by the microscopic structure of the electrodes as well as the convection of the electrolyte.

### 3.2 Electrochemical impedance spectroscopy

**Fig. 2:**
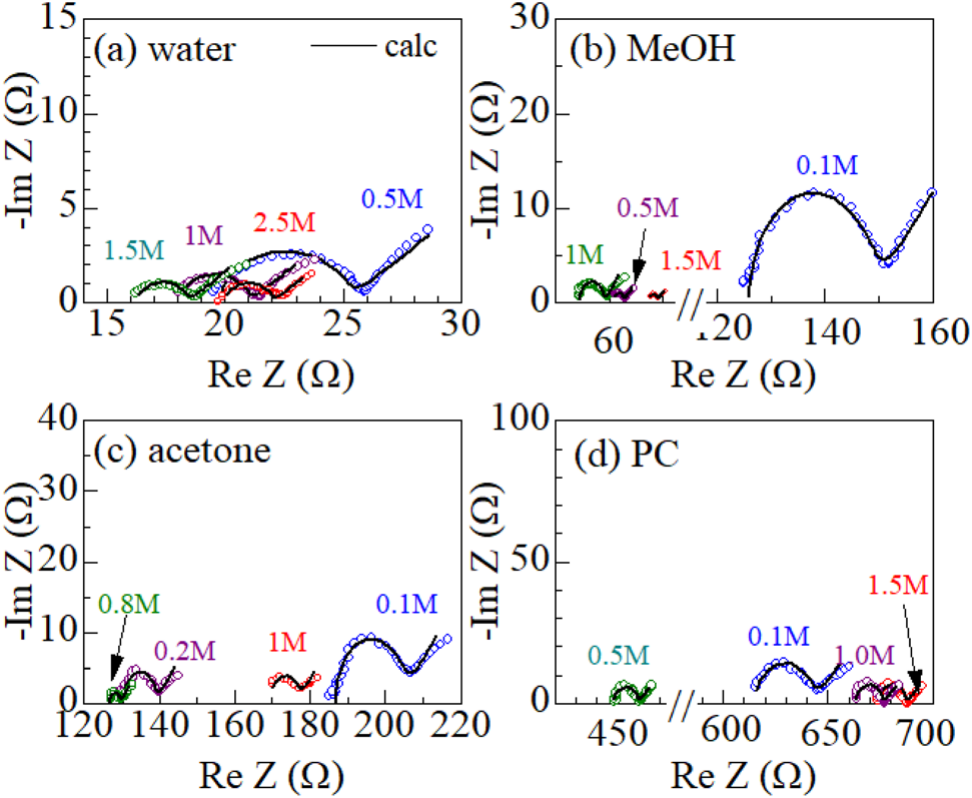
Cole–Cole plots of complex impedance for several solutions containing dissolved Fe^2+^/Fe^3+^ at 298 K: (a) water, (b) MeOH, (c) acetone, and (d) PC. The solid curves are the results of least-squares fits with a Randles equivalent circuit composed of *R*_s_, *R*_ct_, *C*_d_, and *Z*_ω_ (see text).

Fig. 2 shows examples of the Cole–Cole plots of complex impedance for several solutions containing dissolved Fe^2+^/Fe^3+^ at 298 K: (a) water, (b) MeOH, (c) acetone, and (d) PC. The Cole–Cole plot of 0.1 M MeOH solution (Fig. 2(b)) shows a prototypical shape. The plot shows a semicircle on the left side and a straight line with an inclination of 45° on the right side. The resistances on the left and right sides of the semicircle correspond to *R*_s_ and *R*_s_ + *R*_ct_, respectively. The intersection of the straight line with the horizontal axis corresponds to *R*_s_ + *R*_ct_ − 2*A*_W_^2^*C*_d_. Similar behaviors are also observed in the other solutions. The solid curves in Fig. 1 are the results of least-squares fits with a Randles equivalent circuit composed of *R*_s_, *R*_ct_, *C*_d_, and *Z*_ω_. The Randles equivalent circuit well reproduces the observed complex impedance. Thus, we obtained *R*_s_, *R*_ct_, *C*_d_, and *A*_W_. We further evaluate *R*_dif_ (= *R*_tot_ − *R*_s_ − *R*_ct_) with the use of *R*_tot_. The obtained *R*_s_, *R*_ct_, *R*_dif_, and *A*_W_ values are listed in Table 2.

*A*_W_ is expected to have a strong correlation with *R*_dif_ because *Z*_ω_ [= *A*_W_(*ω*^−1/2^ − i*ω*^−1/2^)] describes the diffusion process of the reactants/products. We calculated the correlation coefficient *X* between *A*_W_ and *R*_dif_ (= *R*_tot_ − *R*_s_ − *R*_ct_) for each solution system; *X* = 0.976 for water, 0.995 for MeOH, 0.980 for acetone, and 0.988 for PC. The positive correlation (*X* ≥ 0.976) between *A*_W_ and *R*_dif_ strongly supports the correctness of our evaluation method of *R*_dif_.

### 3.3 Concentration dependence of resistivity components

**Fig. 3:**
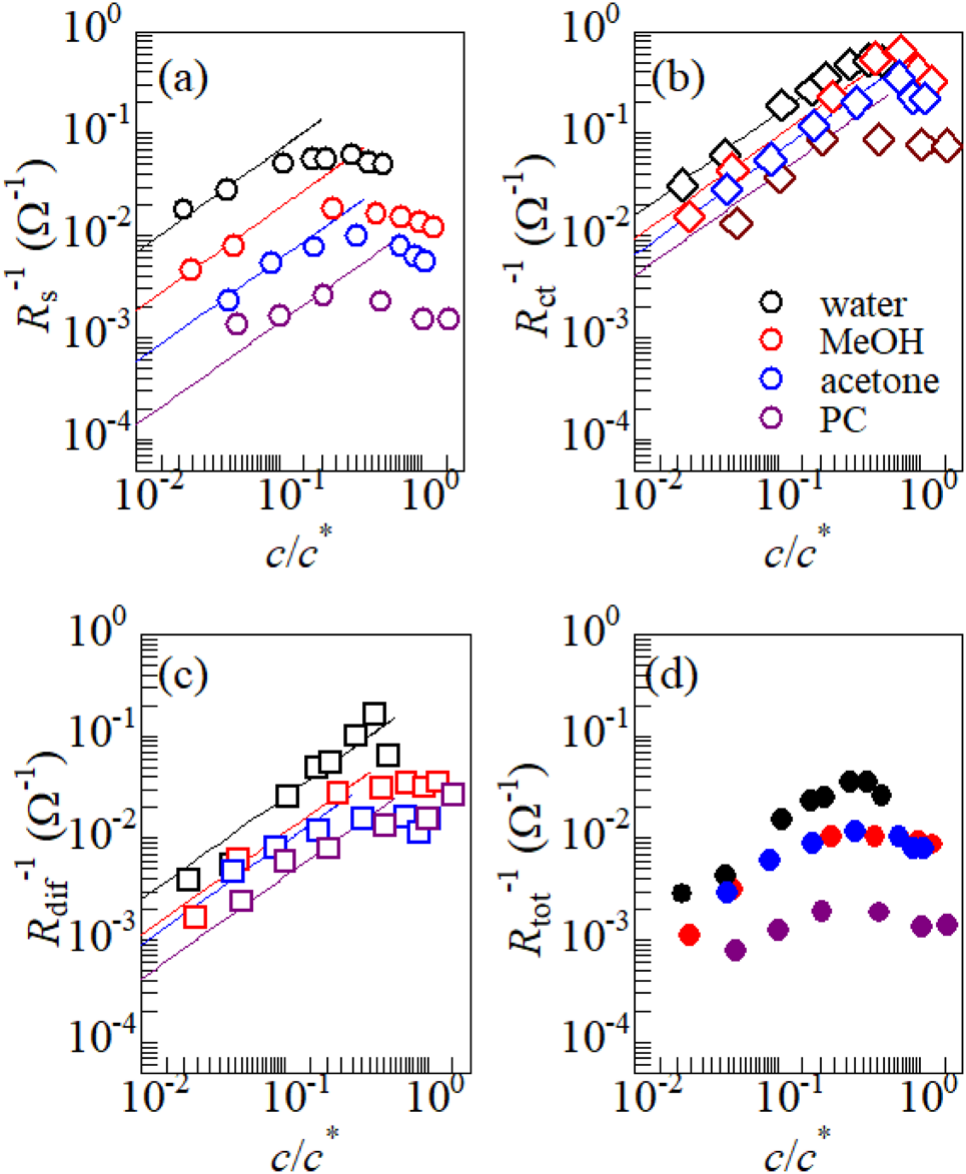
Solute concentration (*c*) dependence of (a) *R*_s_^−1^, (b) *R*_ct_^−1^, (c) *R*_dif_^−1^, and (d) *R*_tot_^−1^ in several solutions containing dissolved Fe^2+^/Fe^3+^ at 298 K. The horizontal axis is normalized by the critical concentration c* of each solvent. The straight lines in (a), (b), and (c) represent the linear relation with 
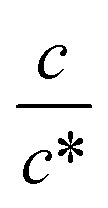
.

Fig. 3 shows the *c*-dependence of (a) *R*_s_^−1^, (b) *R*_ct_^−1^, (c) *R*_dif_^−1^, and (d) *R*_tot_^−1^ in several solutions containing dissolved Fe^2+^/Fe^3+^ at 298 K. For the convenience of explanation, the horizontal axis is normalized by the critical concentration c* of each solvent. At *c* = c*, all solvent molecules are on average coordinated with Fe ions.

First, let us examine the solvent dependence of *R*_s_^−1^, *R*_ct_^−1^, and *R*_dif_^−1^. Significant solvent dependence is observed for (a) *R*_s_^−1^. *R*_s_^−1^ values at 
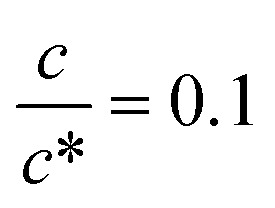
 are 69.5 × 10^−3^ Ω^−1^ in water, 17.1 × 10^−3^ Ω^−1^ in MeOH, 6.7 × 10^−3^ Ω^−1^ in acetone, and 1.4 × 10^−3^ Ω^−1^ in PC. The *R*_s_^−1^ values in organic solutions are much smaller than those in aqueous solutions. In contrast, *R*_ct_^−1^ and *R*_dif_^−1^ have relatively small solvent dependence. In this sense, reducing *R*_s_ is effective to reduce *R*_tot_ in organic solution. Shortening *d* is especially effective because *κ* (≈ 0.2 W K^−1^ m^−1^) of an organic solvent is much smaller than *κ* (= 0.6 W K^−1^ m^−1^) of water. Reflecting the small *κ* in organic solvent, a sufficient Δ*T* is expected between the electrodes, even in the cell with smaller *d*.

Next, let us investigate the 
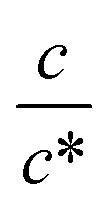
 dependence of *R*_s_^−1^, *R*_ct_^−1^, and *R*_dif_^−1^. In the small 
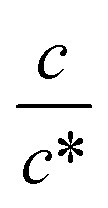
 region, *R*_s_^−1^ increases linearly with 
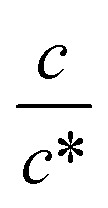
 as indicated by the straight lines in Fig. 3(a). The increase in *R*_s_^−1^ is due to the increase in the number (∝ *c*) of charge carriers, such as Fe^2+^ and Fe^3+^. Upon further increasing 
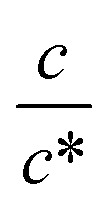
 beyond ∼0.3, *R*_s_^−1^ begins to decrease with 
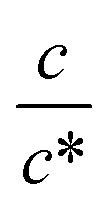
. Similarly, in the small 
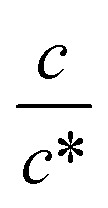
 region, *R*_ct_^−1^ and *R*_dif_^−1^ increase linearly with 
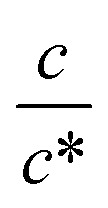
 as indicated by the straight lines in Fig. 3(b) and (c), respectively. The increase in *R*_ct_^−1^ and *R*_dif_^−1^ is due to the increase (∝ *c*) of reactant/product concentration, *i.e.*, Fe^2+^/Fe^3+^. Upon further increasing 
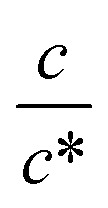
 beyond ∼0.5, *R*_ct_^−1^ and *R*_dif_^−1^ begin to saturate. The saturation of *R*_ct_^−1^ can be ascribed to the finite reaction number (*N*_reaction_) per unit time at the electrode surface. The redox reaction cannot keep up with the supply of reactants when the number (*N*_reactant_ ∝ *c*) of reaching reactants per unit time exceeds *N*_reaction_. In such a region, *N*_reaction_ becomes the rate-determining factor for the charge-transfer current *J*_ct_, and hence, *R*_ct_^−1^. As a result, *R*_ct_ becomes constant at sufficiently large *c*.

## 4 Discussion

### 4.1 Concentration dependence of *R*_s_

**Fig. 4:**
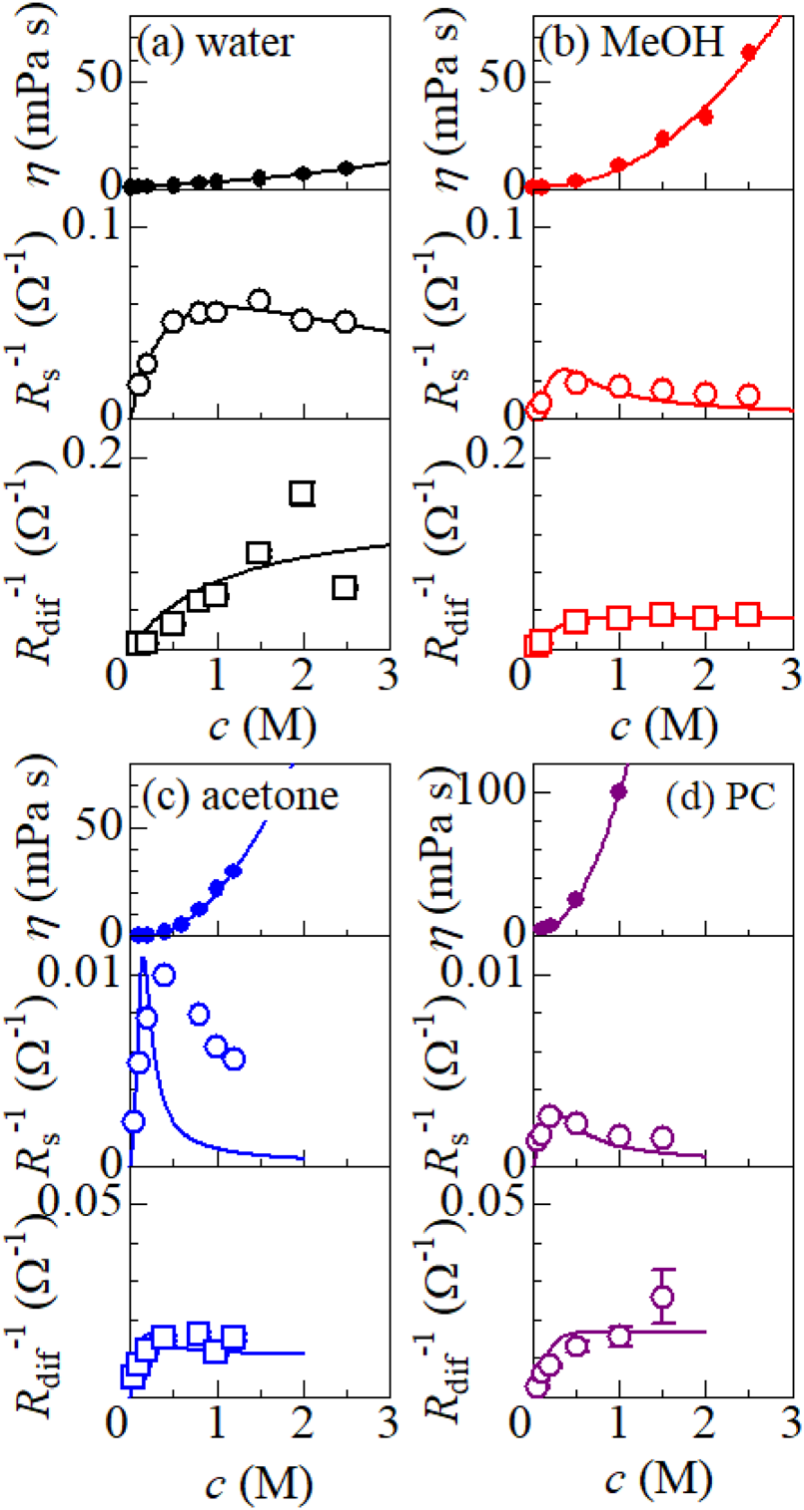
(a) Viscosity *η* (top), *R*_s_^−1^ (middle), *R*_dif_^−1^ (bottom) in aqueous solutions containing dissolved Fe^2+^/Fe^3+^ at 298 K against solute concentration *c*. (b) *η*, *R*_s_^−1^, and *R*_dif_^−1^ in MeOH solutions containing dissolved Fe^2+^/Fe^3+^ at 298 K against *c*. (c) *η*, *R*_s_^−1^, and *R*_dif_^−1^ in acetone solutions containing dissolved Fe^2+^/Fe^3+^ at 298 K against *c*. (d) *η*, *R*_s_^−1^, and *R*_dif_^−1^ in PC solutions containing dissolved Fe^2+^/Fe^3+^ at 298 K against *c*. The solid curves in the upper panels are the results of least-squares fits with a quadratic function. The solid curves in the middle panels are the results of least-squares fits with the 
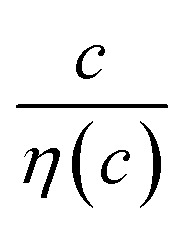
 function. The solid curves in the bottom panels are the results of least-squares fits with the 
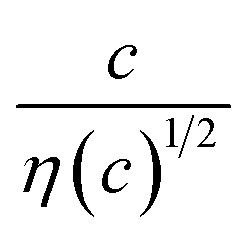
 function.

Now, let us discuss the solution parameters that determine *R*_s_. 
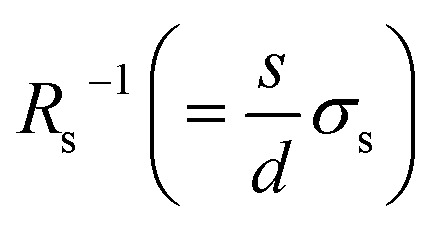
 is expressed as 
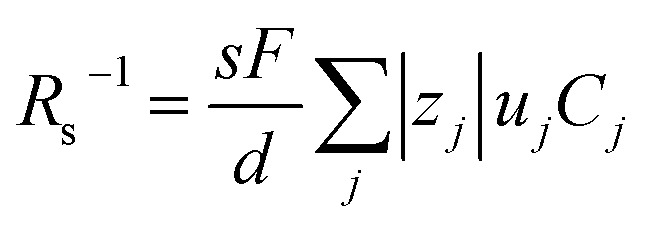
,^23^ where *F*, *z*_*j*_, *u*_*j*_, and *C*_*j*_ are the Faraday constant, charge number, mobility, and molar concentration of the *j*-th ion, respectively. By substituting 
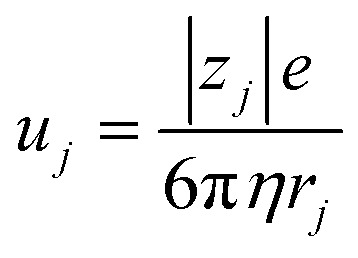
, we obtain 
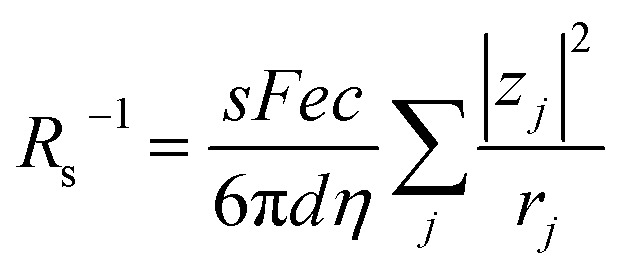
. Note that *C*_Fe^2+^_ = *C*_Fe^3+^_ = *c* in the present solutions. By assuming 
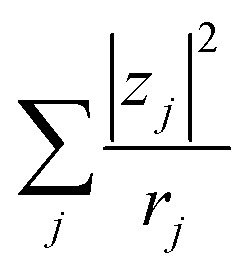
 is independent of *c* in each solution, we obtain the simple relation 
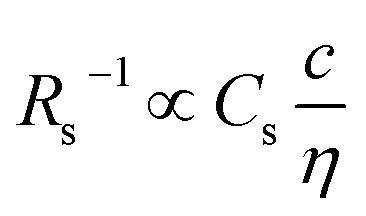
, where *C*_s_ is a constant. The top panels of Fig. 4(a)–(d) show *η* of each solution against *c*. The *η* values were evaluated at 298 K using a sine-wave vibro viscometer (SV-10; A&D Company Limited). In all solutions, *η* increases nonlinearly with *c*. The solid curves are the results of least-squares fits with a quadratic function. With use of the quadratic function *η*(*c*), the empirical formula, 
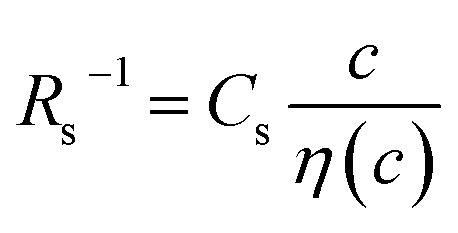
, can be calculated.

The middle panels of Fig. 4(a)–(d) show comparisons between observed *R*_s_^−1^ (open circles) and empirical formula (solid curves) against *c*. We note that there is only one fitting parameter (*C*_s_) to adjust the magnitude but no parameter to adjust the shape. Nevertheless, the curve reproduces the observed *R*_s_^−1^ well, except for (c) acetone solution. In Table 3, we listed *C*_s_. Except for the acetone solution, the solvent dependence of *C*_s_ is rather small, falling between 0.104 mPa s M^−1^ Ω^−1^ and 0.183 mPa s M^−1^ Ω^−1^. This is probably because the *r* value does not change greatly depending on the solvent.

**Table 3:** Coefficients (*C*_s_ and *C*_dif_) of empirical formulas, 
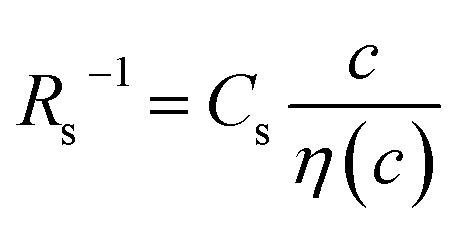
 and 
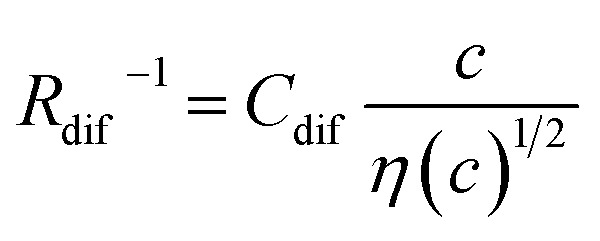
, determined by least-squares fits with the observed data. MeOH and PC represent methanol and propylene carbonate, respectively

Solvent	*C*_s_ (mPa s M^−1^ Ω^−1^)	*C*_dif_ (mPa^1/2^ s^1/2^ M^−1^ Ω^−1^)
Water	0.183	0.125
MeOH	0.132	0.099
Acetone	0.019	0.053
PC	0.104	0.166

In (c) acetone solution, the shape of the *c*–*R*_s_^−1^ plot (open circles) is qualitatively different from the shape of the empirical formula (solid curve). In the region of *c* ≥ 0.5, the empirical formula results decrease steeply while the observed *R*_s_^−1^ decreases slowly. If *C*_s_ is set to ∼0.1, the agreement between the calculated and observed values is improved in the region of *c* ≥ 0.5 even though the calculated value is much larger in the region of *c* ∼ 0.3. This implies that an additional factor, *e.g.*, the repulsive interaction between Fe ions, suppressed *R*_s_ in the region of *c* ∼ 0.3.

### 4.2 Concentration dependence of *R*_dif_

Finally, let us consider the relationship between *R*_dif_^−1^ and *η*. *R*_dif_^−1^ is proportional to the diffusion current *J*_dif_, which is expressed as 
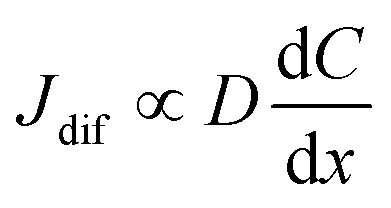
. Replacing the differential with the difference, we get 
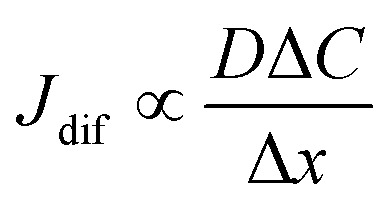
,^23^ where Δ*x* and Δ*C* are the diffusion length and concentration difference between electrode surface and bulk solution, respectively. In one-dimensional diffusion, Δ*x* is expressed as 
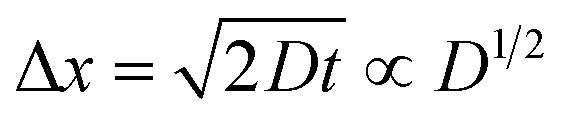
, where *t* is the elapsed time. Then, *J*_dif_ is proportional to *cD*^1/2^ because Δ*C* ∝ *c*. From the Stokes–Einstein equation, we obtain 
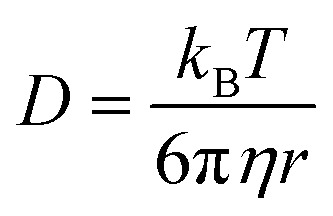
. Finally, we obtain an empirical relation 
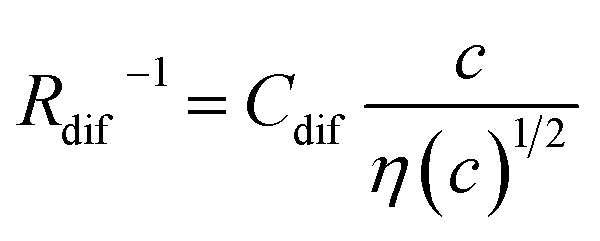
, where *C*_dif_ is a constant. We can calculate the empirical formula with use of the quadratic function *η*(*c*).

The bottom panels of Fig. 4(a)–(d) show comparisons between the observed *R*_dif_^−1^ (open circles) and empirical formula (solid curves) against *c*. We note that there is only one fitting parameter (*C*_dif_) to adjust the magnitude. Nevertheless, the curve reproduces the observed *R*_dif_^−1^ well. In Table 3, we list the *C*_dif_ values. The solvent dependence of *C*_dif_ is rather small, falling between 0.053 mPa^1/2^ s^1/2^ M^−1^ Ω^−1^ and 0.166 mPa^1/2^ s^1/2^ M^−1^ Ω^−1^. This is probably because the *r* value does not change greatly depending on the solvent.

## 5 Conclusions

In conclusion, we investigated the *c*-dependence of *R*_s_, *R*_ct_, and *R*_dif_ in dissolved-Fe^2+^/Fe^3+^-containing aqueous, MeOH, acetone, and PC solutions. We found that the *c*-dependence of *R*_s_ and *R*_dif_ is well reproduced by the empirical formulas 
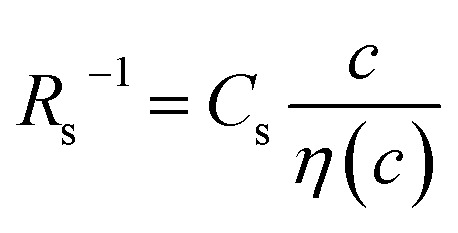
 and 
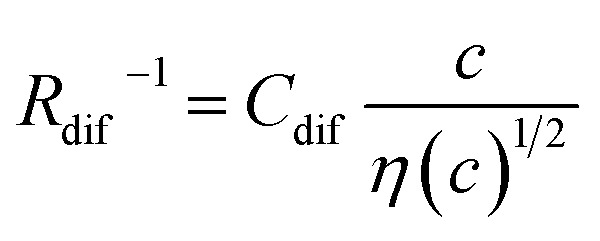
. We further found that the magnitudes of *C*_s_ and *C*_dif_ are nearly independent of the solvent, suggesting that *η* is one of the significant solution parameters that determine *R*_s_ and *R*_dif_. Our findings suggest that *σ* of the electrolyte solution can be increased through reducing *η*.

## Author contributions

Dai Inoue: data curation, formal analysis, and investigation. Yutaka Moritomo: conceptualization, supervision, writing – original draft, and writing – review & editing.

## Conflicts of interest

There are no conflicts to declare.
